# Nutritional impairment, psychological health and quality of life among older adults with advanced cancer: A secondary analysis of a randomized clinical trial

**DOI:** 10.1002/cam4.7348

**Published:** 2024-06-19

**Authors:** Surbhi Singhal, Ying Wang, Zhaoyang Qin, Derick R. Peterson, Richard F. Dunne, Eva Culakova, Judith O. Hopkins, Natalia Melnyk, Adedayo Onitilo, Valerie Targia, Supriya Mohile, Kah Poh Loh

**Affiliations:** ^1^ Division of Hematology/Oncology, Department of Medicine University of California Davis Sacramento California USA; ^2^ Department of Public Health Sciences University of Rochester Medical Center Rochester New York USA; ^3^ Department of Biostatistics and Computational Biology University of Rochester Medical Center Rochester New York USA; ^4^ Division of Hematology and Oncology James P. Wilmot Cancer Institute, University of Rochester Medical Center Rochester New York USA; ^5^ Department of Surgery, Supportive Care in Cancer Unit University of Rochester Rochester New York USA; ^6^ Novant Health Cancer Institute/Southeast Clinical Oncology Research Consortium Winston‐Salem North Carolina USA; ^7^ Delaware/Christiana Care NCI Community Oncology Research Program (NCORP) Newark Delaware USA; ^8^ Wisconsin NCORP Marshfield Wisconsin USA; ^9^ Stakeholders for the Care and Research of Oncology Elders (SCOREBoard) Advisory Committee Duarte USA

**Keywords:** nutritional impairment, older adults, psychological health, quality of life

## Abstract

**Background:**

Nutritional impairment is associated with treatment toxicity and worse overall survival in patients with cancer. We aimed to (1) evaluate the association of nutritional impairment with psychological health and quality of life (QOL) and (2) examine which measures of nutrition had the strongest association with psychological health and QOL among older adults receiving cancer treatment with palliative intent.

**Methods:**

This secondary analysis was performed on baseline data from a nationwide cluster randomized clinical trial (ClinicalTrials.gov identifier: NCT02107443; PI: Mohile). Adults age ≥70 with advanced cancer and ≥1 geriatric assessment (GA) impairment were enrolled from 2014 to 2017. In line with geriatric oncology standards, we defined nutritional impairment as Mini Nutritional Assessment Short Form (MNA‐SF) ≤11, body mass index (BMI) <21 kg/m^2^, or >10% involuntary weight loss in the past 6 months. We conducted multivariable linear regressions to evaluate the association of nutritional impairment with each measure of psychological health and QOL: Geriatric Depression Scale (GDS‐15, range 0–15), Generalized Anxiety Disorder‐7 (GAD‐7, range 0–21), NCCN Distress Thermometer (NCCN DT, range 0–10), and Functional Assessment of Cancer Therapy‐General (FACT‐G, range 0–108). Analyses were adjusted for patient demographics, clinical characteristics, and GA.

**Results:**

Among 541 patients, the mean age was 77 (range 70–96) and 60% had nutritional impairment. Mean baseline scores: GDS‐15 3.1 (SD 2.7), GAD‐7 2.9 (SD 4.0), NCCN DT 2.9 (SD 2.7), and FACT‐G 80 (SD 15). In the adjusted model, compared to those with no nutritional impairment, older adults with nutritional impairment had greater depression (*β* = 0.79, 95% CI 0.36–1.23) and anxiety severity (*β* = 0.86, 95% CI 0.19–1.53), and worse QOL (*β* = −6.31, 95% CI −8.62 to −4.00). Of the measures of nutrition, MNA‐SF ≤11 demonstrated the strongest associations with depression, anxiety, distress, and QOL.

**Conclusion:**

Nutritional impairment is associated with impaired psychological health and worse QOL. Clinicians should use the MNA‐SF to screen older adults for nutritional impairment and offer tailored supportive interventions.

## 1 BACKGROUND

As the U.S. population ages, the number of older adults with cancer is projected to rise between 2015 and 2050, with the largest percentage increase occurring among adults ≥75 years of age.^1^ Older adults are at risk for malnutrition, which is characterized by diminished intake or uptake of nutrition that can lead to weight loss and decreased physical and mental functioning.^2^, ^3^, ^4^, ^5^ While malnutrition is thought to be partially related to the normal aging process, it is a complex and multifactorial process that may include age‐related anorexia with insufficient dietary intake, concurrent cognitive decline, and decreased socioeconomic status, all of which can be accelerated by the presence of comorbid medical conditions.^6^, ^7^, ^8^ Older adults with cancer are especially vulnerable to the development of malnutrition, given the cancer, its treatment, and the generally high prevalence of comorbid conditions in this population.^9^, ^10^ Malnutrition leads to multiple deleterious consequences in older adults with cancer, including a higher risk for chemotherapy toxicity and reduced overall survival.^11^, ^12^

The American Society of Clinical Oncology (ASCO) recognizes nutrition as a key component of the geriatric assessment (GA) and the personalized treatment and supportive care plan that can follow.^13^, ^14^ To identify an impairment in nutritional status and screen for malnutrition among older adults with cancer, clinicians can evaluate patients by calculating body mass index (BMI) or percent weight loss, or by utilizing a validated patient‐reported outcome (PRO) instrument.^13^ Like malnutrition, nutritional impairment is also associated with multiple adverse outcomes and the tools described above can be readily accessible in the electronic chart or easily administered in minutes. Specifically, low BMI is associated with a higher risk for chemotherapy toxicity and a lower overall survival among adults with cancer,^15^, ^16^, ^17^, ^18^ while unintentional weight loss among adults with cancer in the 6 months prior to chemotherapy has been associated with lower chemotherapy response rates, decreased performance status, and lower overall survival.^19^, ^20^ The Mini Nutritional Assessment Short Form (MNA‐SF) is a six‐item PRO instrument that combines BMI and weight loss with additional questions about food intake, function, and comorbid conditions to screen for nutritional impairment.^21^ It has confirmed validity as a nutritional impairment screening tool in studies conducted in a general population of older adults.^22^, ^23^

Quality of life (QOL) is an important outcome among older adults, who often prioritize preserving or improving QOL over length of life.^24^ Prior studies demonstrated that poor nutrition is associated with worse QOL, but these studies were restricted to a single cancer type or single study site.^5^, ^25^, ^26^ We conducted a secondary analysis of a national clinical trial to evaluate the association of nutritional impairment—as identified by various screening tools—with psychological health and QOL among older adults with a wide variety of advanced solid tumors or lymphomas who were receiving cancer treatment with palliative intent. To explore the utility of the various measures of nutritional impairment (MNA‐SF, BMI, and weight loss), we examined which measure of nutrition had the greatest association with psychological health and QOL.

## 2 METHODS

### 2.1 Study design, setting, and participants

We performed a secondary analysis of baseline data from the Improving Communication in Older Cancer Patients and Their Caregivers (COACH) trial (NCT02107443; Principal Investigator: Supriya Mohile). COACH was a nationwide, cluster‐randomized clinical trial conducted within the University of Rochester Cancer Center National Cancer Institute Community Oncology Research Program (NCORP). The primary study enrolled patients who were age ≥70 years, diagnosed with incurable lymphoma or solid tumor, receiving or planning to receive noncurative cancer treatment (systemic therapy and/or radiation therapy), and had ≥1 impaired GA domain between October 29, 2014 and April 28, 2017. The full details of the trial design were previously published.^27^ A total of 541 patients from 31 community oncology practices enrolled in the parent study. All patients and oncologists provided written informed consent to their participation in COACH and subsequent secondary analyses. The University of Rochester Research Subjects Review Board and the review boards of the participating NCORP affiliates approved this study.

### 2.2 Independent variable: Nutritional impairment

We defined nutritional impairment using an aggregate definition: MNA‐SF^21^ ≤11, BMI <21 kg/m,^2^ or >10% involuntary weight loss in the past 6 months. This definition is consistent with the definition used in the parent study.^27^ The MNA‐SF assesses patients' food intake, weight loss in the past 3 months, BMI, mobility, psychological stress, acute disease, and cognitive impairment.^21^ The MNA‐SF is scored from 0 to 14 points, with scores ≤11 indicating nutritional impairment.^21^, ^28^

### 2.3 Dependent variables: Psychological health and QOL

Psychological health was assessed using validated instruments including the Geriatric Depression Scale (GDS‐15),^29^ the Generalized Anxiety Disorder 7‐Item Scale (GAD‐7),^30^ and the National Comprehensive Cancer Network distress thermometer (NCCN DT).^31^ QOL was assessed using the Functional Assessment of Cancer Therapy‐General (FACT‐G).^32^ The GDS‐15 is a 15‐item PRO that is scored from 0 to 15, where a score ≥5 suggests depression, and higher scores indicate greater depression severity. The minimal clinically important difference (MCID) in GDS‐15 is 1.2 points.^33^ The GAD‐7 is a seven‐item PRO that is scored from 0 to 21, where higher scores indicate greater anxiety severity and the MCID is three points.^34^ The NCCN DT requests patients to rate their distress from a score of 0 to 10, where a score of 10 indicates extreme distress. While no MCID data exist for the NCCN DT, experts recommend a score of ≥4 to identify clinically elevated distress.^35^ The FACT‐G includes 27 items across four domains: physical well‐being, social/family well‐being, emotional well‐being, and functional well‐being. The responses to these items are translated into a score from 0 to 108, where higher scores indicate better QOL. The MCID in FACT‐G is five to six points.^36^

### 2.4 Covariates

Patient‐reported demographics (age, sex, race, education, annual income) and clinical characteristics (cancer type, cancer stage, planned cancer treatment) were collected during the parent study.^27^ As previously described,^27^ all patients completed a GA that, in addition to nutrition and psychological health, encompassed the following six domains: (1) physical performance (Short Physical Performance Battery,^37^ Timed Up and Go,^38^ Older Americans Resources and Services [OARS] physical health,^39^ falls in the last 6 months^40^), (2) functional status (Katz Index of Independence in Activities of Daily Living,^41^ OARS Instrumental Activities of Daily Living^39^), (3) polypharmacy, (4) OARS comorbidity,^39^ (5) cognition (Short Blessed Orientation–Memory–Concentration Test,^42^ Mini‐Cog^43^), and (6) OARS medical social support.^39^ Patients were considered to have at least one impaired GA domain if at least one measure of that domain was impaired.

### 2.5 Statistical analyses

We categorized patients as having nutritional impairment versus no nutritional impairment using the aggregate definition of nutritional impairment defined above. Descriptive statistics were used to summarize patient demographics, clinical characteristics, GA domains, psychological health and QOL. We used two‐sample *t*‐tests or Mann–Whitney *U* test for continuous variables and the chi‐squared test or Fisher's exact tests for categorical variables to compare patients' characteristics, psychological health, and QOL between the two groups.

We conducted separate multivariable linear regressions to evaluate the associations of nutritional impairment (using the aggregate definition) with GDS‐15, GAD‐7, NCCN DT, and FACT‐G. Each multivariable model was adjusted for patient‐reported demographics, clinical characteristics, and GA domains, which were selected a priori as potential confounders.^5^, ^44^ We used a correlation matrix to detect multicollinearity and found insufficient evidence of multicollinearity among covariates. To detect possible nonlinear relationships, we first tested the continuous variable (i.e., age) in the multivariable model using a piecewise linear spline and found insufficient evidence of nonlinearity; we therefore treated age as a continuous variable.

To address missing data among the dependent variables, we used mean imputation, replacing a missing response with the mean of the nonmissing responses. For GDS‐15 and GAD‐7, mean imputation was applied when there were at least ten responses and six responses, respectively. For NCCN DT, no imputation was conducted since it is a single‐item measure. For FACT‐G, we conducted mean imputation when greater than 50% of the items were answered in the subscale (e.g., a minimum of 4 of 7 items). After imputation, there were no missing values for GDS‐15, no missing values for GAD‐7, six missing values for NCCN DT, and 15 missing values for FACT‐G. These missing values represented <3% of the total sample and were therefore excluded from the analysis.

Given the different measures (MNA‐SF, BMI, and weight loss) within the aggregate nutritional impairment definition have been previously validated or associated with poor outcomes in adults with cancer, we then considered each measure individually. To determine which measure of nutritional impairment had the strongest association with psychological status and QOL, we additionally defined nutritional impairment as: (1) MNA‐SF ≤11 (yes versus no), (2) BMI <21 kg/m^2^ (yes versus no), and (3) >10% involuntary weight loss in the past 6 months (yes versus no), and (4) a three‐level categorical variable (no nutritional impairment [reference group], MNA‐SF ≤11 only [no impairment in weight loss or BMI], and BMI <21 kg/m^2^ or >10% involuntary weight loss [with or without MNA‐SF ≤11]). These three levels were selected for two reasons. First, based on the sample size of three impaired components, the current categorization ensures we have enough statistical power to conduct multivariable regressions. Second, the current categorization enables us to compare the strength of associations with psychological health and QOL between readily available measures in clinical settings (i.e., BMI, weight) and less available measure (i.e., MNA‐SF). Associations of nutritional impairment defined using these four methods with psychological health and QOL were separately examined using similar linear regression models described above.

Statistical significance was set as a 2‐tailed *p* < 0.05. Statistical analyses were conducted using SAS software, Version 9.4 of the SAS system.^45^

## 3 RESULTS

### 3.1 Patient characteristics

**FIGURE 1:**
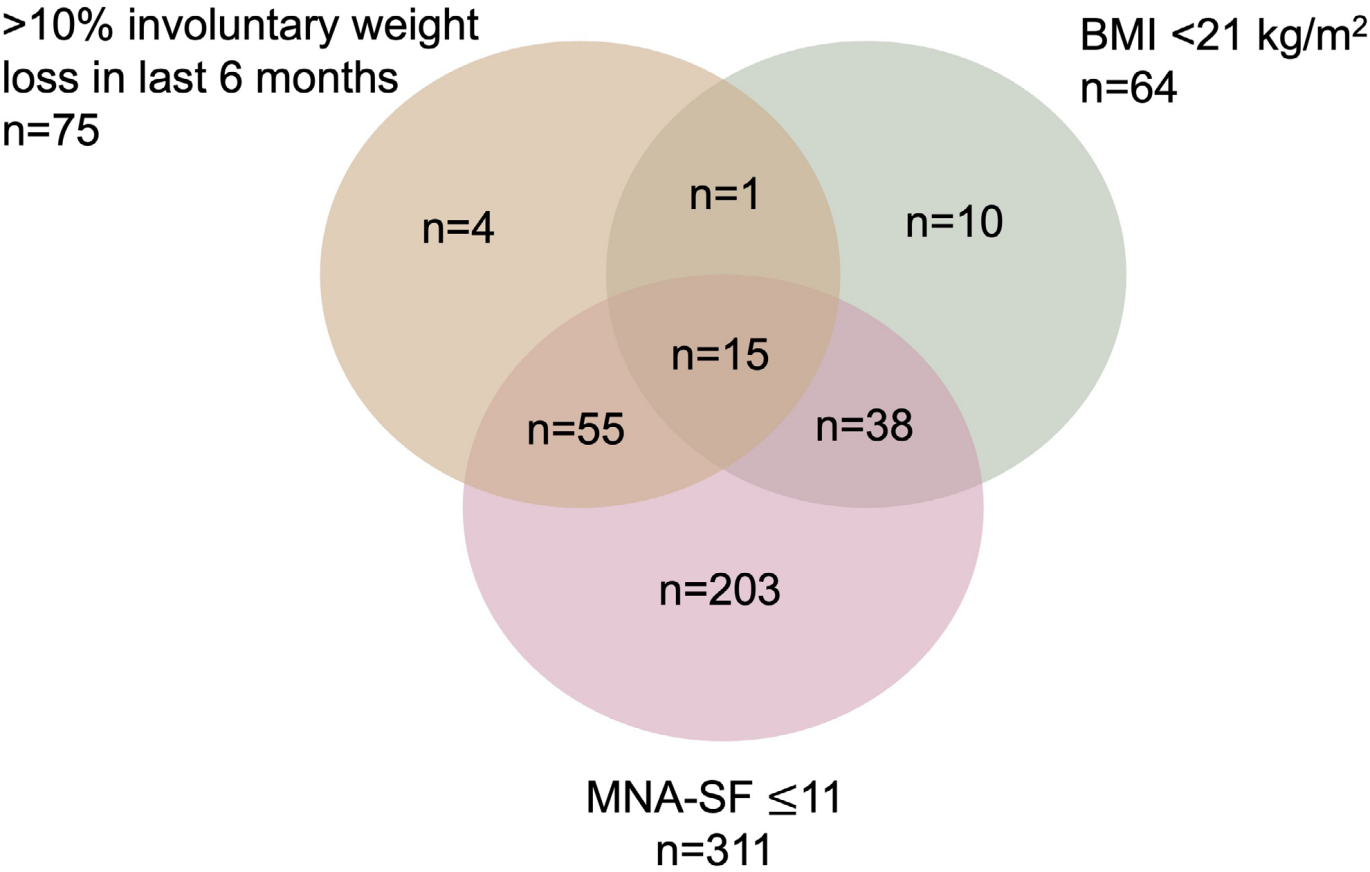
Venn diagram of different measures related to nutritional impairment (*n* = 326). Among the 326 patients with nutritional impairment, MNA‐SF ≤11 was the most common impaired measure of the nutrition domain (*n* = 311, 95%), followed by >10% weight loss in the last 6 months (*n* = 75, 23%), and BMI <21 kg/m^2^ (*n* = 64, 20%). Only 15 patients (6%) had impairments in all three measures of the nutrition domain. BMI, body mass index; MNA‐SF, Mini Nutritional Assessment Short Form.

All 541 patients enrolled in the COACH trial were included in this secondary analysis **(**Table 1 **)**. Patients had a mean age of 77 years (standard deviation [SD] 5.2, range 70–96 years), and 326 (60%) met criteria for nutritional impairment. Of the entire cohort, 486 (90%) were white, 279 (52%) had some college education or above, and 480 (89%) had stage IV disease. Patients who had nutritional impairment were more likely to have gastrointestinal cancers, treatment plans that included chemotherapy, and impaired functional status on GA **(**Table 1 **).** Mean baseline GDS‐15 was 3.1 (standard deviation [SD] 2.7), GAD‐7 was 2.9 (SD 4.0), NCCN DT was 2.9 (SD 2.7), and FACT‐G was 80 (SD 15). Among the 326 patients with nutritional impairment, MNA‐SF ≤11 was the most common impaired measure of the nutrition domain (*n* = 311, 95%), followed by >10% weight loss in the last 6 months (*n* = 75, 23%), and BMI <21 kg/m^2^ (*n* = 64, 20%). Only 15 patients (6%) had impairments in all three measures of the nutrition domain (Figure 1).

**TABLE 1:** Patient characteristics.

Characteristics	Total (*N* = 541) *n* (%)	Nutritional impairment (*n* = 326) *n* (%)	No nutritional impairment (*n* = 215) *n* (%)	*p*‐Value
Patient‐reported demographics				
Age in years, mean (SD)	77 (5.2)	77 (5.3)	77 (5.1)	0.57
70–79	401 (74.1)	246 (75.5)	155 (72.1)	0.64
80–99	127 (23.5)	72 (22.1)	55 (25.6)	
> =90	12 (2.2)	7 (2.1)	5 (2.3)	
Missing	1 (0.2)	1 (0.3)		
Sex, *n* (%)				
Female	264 (48.8)	163 (50.0)	101 (47.0)	0.47
Male	276 (51.0)	162 (49.7)	114 (53.0)	
Missing	1 (0.2)	1 (0.3)		
Race, *n* (%)				
White	486 (89.8)	295 (90.5)	191 (88.8)	0.78
Black	40 (7.4)	22 (6.7)	18 (8.4)	
Other*	15 (2.8)	9 (2.8)	6 (2.8)	
Education, *n* (%)				
Less than High school	66 (12.2)	44 (13.5)	22 (10.2)	0.50
High school graduate	195 (36.0)	117 (35.9)	78 (36.3)	
Some college or above	279 (51.6)	164 (50.3)	115 (53.5)	
Missing	1 (0.2)	1 (0.3)		
Income, *n* (%)				
≤$50,000	265 (49.0)	158 (48.5)	107 (49.8)	0.62
>$50,000	164 (30.3)	96 (29.4)	68 (31.6)	
Declined to answer	109 (20.1)	70 (21.5)	39 (18.1)	
Missing	3 (0.6)	2 (0.6)	1 (0.5)	
Clinical characteristics				
Cancer type, *n* (%)				
Breast	69 (12.8)	35 (10.7)	34 (15.8)	**0.02**
Gastrointestinal	138 (25.5)	95 (29.1)	43 (20.0)	
Genitourinary	79 (14.6)	39 (12.0)	40 (18.6)	
Gynecologic	34 (6.3)	18 (5.5)	16 (7.4)	
Lung	140 (25.9)	92 (28.2)	48 (22.3)	
Lymphoma	41 (7.6)	21 (6.4)	20 (9.3)	
Other	39 (7.2)	25 (7.7)	14 (6.5)	
Missing	1 (0.2)	1 (0.3)		
Cancer stage, *n* (%)				
III	47 (8.7)	27 (8.3)	20 (9.3)	0.74
IV	480 (88.7)	289 (88.7)	191 (88.8)	
Other	13 (2.4)	9 (2.8)	4 (1.9)	
Missing	1 (0.2)	1 (0.3)		
Chemotherapy in treatment plan, *n* (%)				
Yes	369 (68.2)	241 (73.9)	128 (59.5)	**<0.01**
No	170 (31.4)	83 (25.5)	87 (40.5)	
Missing	2 (0.4)	2 (0.6)		
Targeted therapy in treatment plan, *n* (%)				
Yes	86 (15.9)	49 (15.0)	37 (17.2)	0.51
No	454 (83.9)	276 (84.7)	178 (82.8)	
Missing	1 (0.2)	1 (0.3)		
Impaired Geriatric Assessment Domains				
Physical performance, *n* (%)	507 (93.7)	307 (94.2)	200 (93.0)	0.59
Functional status, *n* (%)	319 (59.0)	208 (63.8)	111 (51.6)	**<0.01**
Polypharmacy, *n* (%)	453 (83.7)	275 (84.4)	178 (82.8)	0.63
Comorbidity, *n* (%)	344 (63.6)	207 (63.5)	137 (63.7)	0.96
Cognition, *n* (%)	180 (33.3)	111 (34.0)	69 (32.1)	0.64
Medical social support, *n* (%)	156 (28.8)	91 (27.9)	65 (30.2)	0.56

*Note:* The bold values denote *p*‐values that are considered statistically significant.

* Other races include American Indian or Alaskan Native, Asian, Native Hawaiian or other Pacific Islander, and greater than one race.

### 3.2 Association of nutritional impairment with psychological health and QOL by aggregate definition of nutritional impairment

On bivariate analysis, compared to patients with no nutritional impairment, those with nutritional impairment were more likely to report greater depression severity (mean GDS‐15: 3.5 versus 2.5, *p* < 0.01), greater anxiety severity (mean GAD‐7: 3.3 versus 2.2, *p* = 0.01), more distress (mean NCCN DT: 3.2 versus 2.5, *p* = 0.01), and worse QOL (mean FACT‐G: 77.5 versus 85.3, *p* < 0.01).

In the adjusted multivariable model, compared to those with no nutritional impairment, older adults with nutritional impairment had greater depression severity (*β* = 0.79, 95% confidence interval [CI] 0.36 to 1.23), greater anxiety severity (*β* = 0.86, 95% CI 0.19 to 1.53), and worse QOL (*β* = −6.31, 95% CI −8.62 to −4.00) **(**Table 2 **)**. There was insufficient evidence of an association between nutrition and distress (*β* = 0.36, 95% CI −0.09 to 0.82).

**TABLE 2:** Multivariable Linear Analysis for Psychological Health and Quality of Life.

Characteristics	Geriatric Depression Scale‐15 *β* coefficient (95% CI)	Generalized Anxiety Disorder‐7 *β* coefficient (95% CI)	NCCN Distress Thermometer *β* coefficient (95% CI)	Functional Assessment of Cancer Therapy‐General *β* coefficient (95% CI)
Nutritional impairment^a^ (vs. no nutritional impairment)	0.79 (0.36 to 1.23)	0.86 (0.19 to 1.53)	0.36 (−0.09 to 0.82)	−6.31 (−8.62 to −4.00)
Age	−0.04 (−0.09 to −0.01)	−0.08 (−0.14 to −0.01)	−0.07 (−0.11 to −0.03)	0.25 (0.02 to 0.47)
Male (vs. female)	0.38 (−0.13 to 0.89)	−0.50 (−1.28 to 0.28)	−0.27 (−0.81 to 0.27)	−3.07 (−5.78 to −0.36)
Race (vs. white)				
Black	−0.16 (−0.99 to 0.68)	−0.20 (−1.48 to 1.07)	−0.70 (−1.58 to 0.19)	2.32 (−2.14 to 6.79)
Other	0.07 (−1.26 to 1.40)	−1.11 (−3.14 to 0.92)	−0.30 (−1.68 to 1.08)	−2.44 (−9.36 to 4.49)
Education (vs. some college or above)				
Less than high school	0.31 (−0.39 to 1.01)	0.83 (−0.24 to 1.91)	0.47 (−0.27 to 1.21)	−0.55 (−4.29 to 3.19)
High school graduate	0.32 (−0.15 to 0.79)	0.65 (−0.07 to 1.36)	0.07 (−0.41 to 0.56)	−0.71 (−3.19 to 1.76)
Annual income (vs. ≤$50,000)				
>$50,000	0.18 (−0.33 to 0.70)	−0.02 (−0.80 to 0.77)	0.13 (−0.41 to 0.67)	1.88 (−0.84 to 4.60)
Declined to answer	0.15 (−0.41 to 0.71)	−0.18 (−1.03 to 0.68)	−0.37 (−0.95 to 0.22)	0.32 (−2.68 to 3.32)
Cancer type (vs. gastrointestinal)				
Breast	0.79 (−0.01 to 1.59)	−0.67 (−1.89 to 0.56)	−0.28 (−1.13 to 0.56)	−2.33 (−6.56 to 1.90)
Genitourinary	0.57 (−0.19 to 1.33)	−0.12 (−1.27 to 1.04)	−0.10 (−0.89 to 0.69)	−1.35 (−5.35 to 2.66)
Gynecologic	0.15 (−0.83 to 1.14)	−0.19 (−1.70 to 1.32)	−0.28 (−1.31 to 0.76)	0.08 (−5.16 to 5.33)
Lung	0.53 (−0.07 to 1.12)	0.13 (−0.78 to 1.04)	0.22 (−0.41 to 0.84)	−2.10 (−5.25 to 1.04)
Lymphoma	0.47 (−0.40 to 1.35)	1.00 (−0.34 to 2.34)	0.04 (−0.88 to 0.95)	4.17 (−0.40 to 8.75)
Other	0.66 (−0.23 to 1.55)	0.66 (−0.70 to 2.03)	−0.02 (−0.95 to 0.91)	−0.98 (−5.76 to 3.80)
Cancer stage (vs. stage IV)				
III	−0.01 (−0.76 to 0.74)	0.37 (−0.78 to 1.52)	−0.19 (−0.98 to 0.60)	−1.43 (−5.37 to 2.51)
Other	0.89 (−0.54 to 2.33)	0.07 (−2.12 to 2.27)	−0.43 (−1.92 to 1.07)	1.04 (−6.46 to 8.54)
Chemotherapy in treatment plan (vs. no)	0.47 (−0.04 to 0.99)	0.55 (−0.23 to 1.33)	0.59 (0.06 to 1.13)	−2.75 (−5.46 to −0.04)
Targeted therapy in treatment plan (vs. no)	−0.14 (−0.73 to 0.45)	−0.14 (−1.04 to 0.76)	−0.23 (−0.84 to 0.39)	−1.23 (−4.35 to 1.89)
Impaired physical performance (vs. no)	0.50 (−0.39 to 1.39)	−0.65 (−2.01 to 0.71)	0.07 (−0.86 to 0.99)	−2.63 (−7.27 to 2.00)
Polypharmacy (vs. no)	−0.18 (−0.76 to 0.41)	0.30 (−0.59 to 1.20)	0.28 (−0.32 to 0.89)	−1.90 (−4.98 to 1.18)
OARS comorbidity (vs. no)	0.90 (0.45 to 1.35)	1.29 (0.60 to 1.98)	0.62 (0.15 to 1.09)	−4.32 (−6.71 to −1.93)
Impaired functional status (vs. no)	1.43 (0.96 to 1.90)	0.70 (−0.02 to 1.41)	0.81 (0.32 to 1.30)	−8.09 (−10.57 to −5.62)
Impaired cognition (vs. no)	0.62 (0.15 to 1.10)	1.28 (0.55 to 2.01)	0.82 (0.33 to 1.32)	−2.56 (−5.09 to −0.04)
Impaired social support (vs. no)	0.21 (−0.27 to 0.69)	0.18 (−0.56 to 0.91)	0.21 (−0.29 to 0.72)	−1.81 (−4.34 to 0.73)

Abbreviations: CI, confidence interval; OARS, Older Americans Resources and Services.

^a^ Nutritional impairment was defined using an aggregate definition: Mini Nutritional Assessment Short Form ≤11, body mass index <21 kg/m^2^, or >10% involuntary weight loss in the past 6 months.

### 3.3 Association of nutritional impairment with psychological health and QOL by different measures of nutritional impairment

**FIGURE 2:**
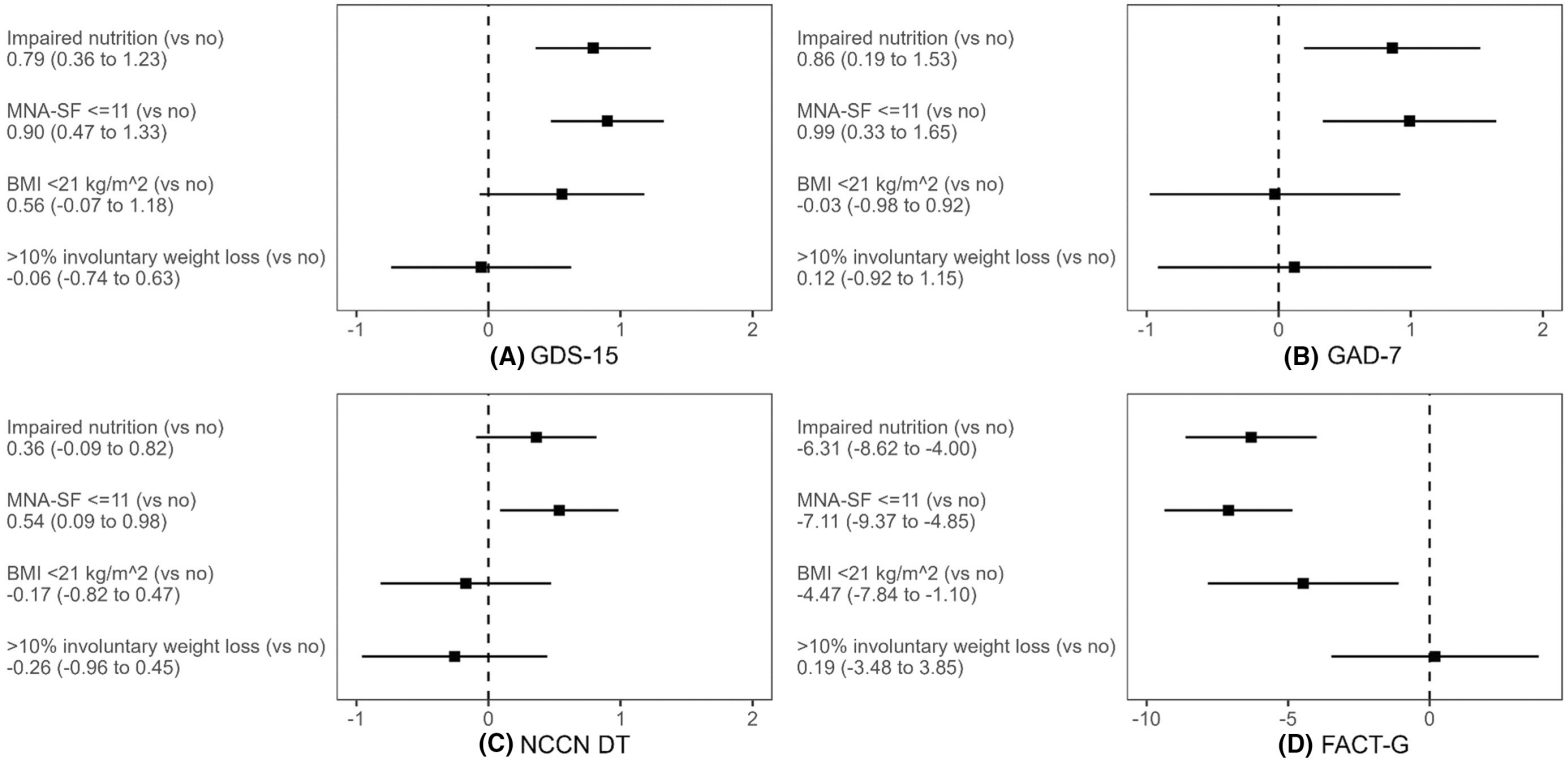
Adjusted association of nutritional impairment by different definitions with (A) depression, (B) anxiety, (C) distress, and (D) quality of life. Each panel presents *four unique* multivariable linear regression models with nutritional impairment respectively defined by: (1) aggregate definition (MNA‐SF ≤11, BMI <21 kg/m^2^, or >10% involuntary weight loss in the past 6 months); (2) MNA‐SF ≤11; (3) BMI <21 kg/m^2^; and (4) >10% involuntary weight loss in the past 6 months. MNA‐SF demonstrated the strongest association with all outcomes of psychological health and quality of life. BMI, body mass index; FACT‐G, Functional Assessment of Cancer Therapy‐General; GAD‐7, Generalized Anxiety Disorder 7‐Item Scale; GDS‐15, Geriatric Depression Scale; NCCN DT, National Comprehensive Cancer Network distress thermometer, MNA‐SF, Mini Nutritional Assessment Short Form.

Figure 2 presents the adjusted associations of nutritional impairment by different definitions ([1] aggregate definition of MNA‐SF ≤11, BMI <21 kg/m^2^, or >10% involuntary weight loss in the past 6 months; [2] MNA‐SF ≤11; [3] BMI <21 kg/m^2^; and [4] >10% involuntary weight loss in the past 6 months) with each of the four outcomes ([1] GDS‐15; [2] GAD‐7; [3] NCCN DT; [4] FACT‐G), respectively. Among these different definitions, MNA‐SF ≤11 demonstrates the strongest associations with depression (*β* = 0.90, 95% CI 0.47 to 1.33), anxiety (*β*=0.99, 95% CI 0.33 to 1.65), distress (*β* = 0.54, 95% CI 0.09 to 0.98), and QOL (*β* = −7.11, 95% CI −9.37 to −4.85). BMI <21 kg/m^2^ was significantly associated with QOL (*β* = −4.47, 95% CI −7.84 to −1.10), but not significantly associated with depression, anxiety, or distress. The associations between weight loss and the four outcomes were not significant.

**FIGURE 3:**
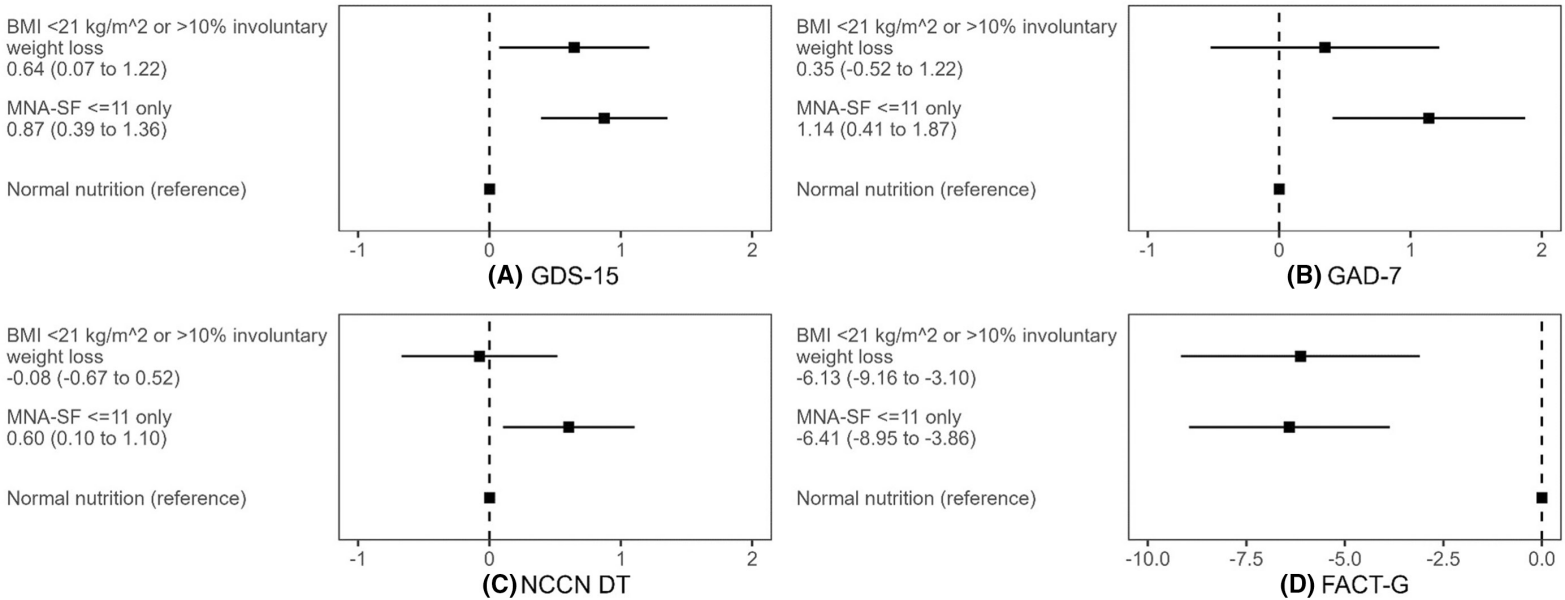
Adjusted association of nutritional impairment defined as a categorical variable with (A) depression, (B) anxiety, (C) distress, and (D) quality of life. Each panel presents a single multivariable linear regression model with nutritional impairment defined as a three‐level categorical variable: (1) no nutritional impairment (reference group); (2) MNA‐SF ≤11 only (no impairment in weight loss or BMI); and (3) BMI <21 kg/m^2^ or >10% involuntary weight loss (with or without MNA‐SF ≤11). BMI, body mass index; FACT‐G, Functional Assessment of Cancer Therapy‐General; GAD‐7, Generalized Anxiety Disorder 7‐Item Scale; GDS‐15, Geriatric Depression Scale; NCCN DT, National Comprehensive Cancer Network distress thermometer; MNA‐SF, Mini Nutritional Assessment Short Form.

Figure 3 depicts the association of nutritional impairment with psychological health and QOL when nutritional impairment is considered a three‐level variable. Compared to no nutritional impairment, MNA‐SF ≤11 (and no impairment in weight loss or BMI) was significantly associated with depression (*β* = 0.87, 95% CI 0.39 to 1.36), anxiety (*β* = 1.14, 95% CI 0.41 to 1.87), distress (*β* = 0.60, 95% CI 0.10 to 1.10), and QOL (*β* = −6.41, 95% CI −8.95 to −3.86). Compared to no nutritional impairment, BMI <21 kg/m^2^ or >10% involuntary weight loss in the past 6 months (regardless of MNA‐SF score) was significantly associated with depression (*β* = 0.64, 95% CI 0.07 to 1.22) and QOL (*β* = −6.13, 95% CI −9.16 to −3.10), but not significantly associated with anxiety or distress.

## 4 DISCUSSION

To our knowledge, this is the first study to characterize the association of impaired nutritional status with psychological health and QOL among older adults with advanced cancers. Nutritional impairment was common and associated with greater depression and anxiety severity and worse QOL. Among the individual measures of nutritional impairment that were examined, only the MNA‐SF was associated with worse psychological health outcomes (GDS‐15, GAD‐7, and NCCN DT) and QOL (FACT‐G).

In this large cohort of older adults with advanced cancer receiving palliative‐intent treatment, 60% met criteria for nutritional impairment. Most of these patients had either gastrointestinal or lung cancers, were receiving cytotoxic chemotherapy, and had impaired physical performance, functional status, comorbid conditions, and/or polypharmacy. Even when adjusting for these impaired geriatric domains, nutritional impairment was independently associated with greater depression severity, greater anxiety severity, and worse QOL. Our finding that older adults with nutritional impairment reported a FACT‐G that on average was 6.31 points lower than the FACT‐G reported by those with no nutritional impairment meets the previously published MCID for FACT‐G, a difference perceived as meaningful by the patient.^36^

Among the measures we used to define nutritional impairment, the MNA‐SF most frequently identified patients with nutritional impairment.^28^ We also found that the MNA‐SF demonstrated the strongest association with impaired psychological health and QOL. Cancer clinicians infrequently use GA tools in clinical practice for older adults with cancer.^46^, ^47^ By assessing food intake and mobility in addition to BMI and weight loss, the MNA‐SF allows oncologists to identify patients at risk for malnutrition as well as poor cancer‐related outcomes that may not be captured when assessing BMI or weight loss alone. The MNA‐SF takes less than 5 min to complete and has a reported sensitivity of 54%–90% and specificity of 61%–88% compared to detailed nutritional assessments among older adults.^28^, ^48^, ^49^ Herein, we provide support for the use of the MNA‐SF to screen for nutritional impairment among older adults with advanced cancers.

Unlike the MNA‐SF, >10% involuntary weight loss in the past 6 months was not associated with depression or anxiety severity, distress, or QOL. A systematic review of studies of adults with multiple cancer types demonstrated that weight loss was associated with worse QOL in 85% of the included reports.^50^ There could be several reasons for our discordant findings. First, unintentional weight loss is very common in older adults in general.^51^, ^52^ It is possible that weight loss alone does not impact QOL in a cancer population of older adults, but rather other factors like preserved mobility and cognition are more valued. Secondly, we adjusted for multiple factors including the GA domains, which may explain our finding that weight loss was not associated with psychological health or QOL compared to the studies in the systematic review. Future larger studies evaluating the relationship between weight loss and QOL would be valuable to clarify whether an association exists between weight loss and QOL among older adults with cancer.

Our finding that nutritional impairment is associated with greater depression and anxiety severity and worse QOL has implications for nutritional screening and supportive care. The European Society for Clinical Nutrition and Metabolism (ESPEN) and ASCO provide clinical practice guidelines to support adults with cancer who have poor nutritional status or cancer cachexia, respectively.^53^, ^54^ These guidelines include referring patients to a registered dietitian, providing dietary advice on minimal total caloric and protein intake, identification and treatment of symptoms impairing food intake, and consideration of oral nutritional supplements. A recent review identified four randomized clinical trials that evaluated the feasibility and efficacy of nutritional interventions among older adults with cancer.^55^ The authors found that nutritional interventions with a dietitian—either by directly interfacing with the patient or indirectly by providing education to the oncologists—resulted in improved QOL. However, there is disparate access to nutrition services in outpatient cancer centers and those with impaired psychological health and QOL may have additional challenges in accessing nutrition services and maintaining an adequate diet.^56^ Furthermore, while the nutritional interventions and guidelines are primarily for patients with gastrointestinal malignancies, in this study we demonstrate that nutritional impairment is common among other populations as well.^57^, ^58^ Given the strong relationship between nutritional impairment with psychological health and QOL demonstrated here, multidimensional interventions that address multiple GA domains, including nutrition, may be particularly useful to improve psychological health and QOL.^59^ Additional studies on nutritional and multidimensional interventions that can be efficiently and effectively integrated into the outpatient cancer centers are needed to support older adults with nutritional impairment to ultimately improve QOL during cancer treatment.

Our study has several strengths. We included a large group of older adults with impaired GA, who historically have been excluded from cancer clinical trials. Furthermore, the included patients were from multiple sites in the community oncology setting, which allowed us to capture patients who might have been missed if the study was conducted at a single academic center. Finally, we included patients with cancers of various types, thereby increasing the generalizability of our findings.

Our study also has several limitations. We evaluated the association of nutritional impairment with psychological health and QOL but did not compare this to a formal malnutrition diagnosis, as identified by the ASPEN/AND criteria.^60^ We also did not collect data on the number or type of prior cancer treatments among the cohort, and whether patients were planning or currently receiving systemic cancer treatments, which could affect the rate of nutritional impairment. Additionally, most patients in the cohort were white with advanced education, which limits the generalizability of our findings. Finally, we conducted a cross‐sectional study using baseline data from the COACH clinical trial, and so are unable to determine if there is a causal relationship between nutritional impairment and psychological health and QOL.

In conclusion, among older adults with advanced cancer, nutritional impairment is common and independently associated with impaired psychological health and QOL. Future work is needed to assess if screening for nutritional impairment with the MNA‐SF, paired with tailored supportive care interventions, improves psychological health and QOL among older adults with advanced cancer.

## AUTHOR CONTRIBUTIONS

**Surbhi Singhal:** Conceptualization (equal); writing – original draft (lead). **Ying Wang:** Formal analysis (lead); writing – original draft (supporting). **Zhaoyang Qin:** Formal analysis (supporting); writing – review and editing (equal). **Derick R. Peterson:** Formal analysis (supporting); writing – review and editing (equal). **Richard F. Dunne:** Writing – review and editing (equal). **Eva Culakova:** Writing – review and editing (equal). **Judith O. Hopkins:** Writing – review and editing (equal). **Natalia Melnyk:** Writing – review and editing (equal). **Adedayo Onitilo:** Writing – review and editing (equal). **Valerie Targia:** Writing – review and editing (equal). **Supriya Mohile:** Conceptualization (equal); writing – review and editing (equal). **Kah Poh Loh:** Conceptualization (equal); writing – original draft (supporting).

## FUNDING INFORMATION

This work was supported by contract 4634 from the PCORI Program (SGM), the National Cancer Institute at the National Institutes of Health (UG1CA189961, R00CA237744 to KPL), the National Institute on Aging at the National Institutes of Health (K24AG056589 and R33AG059206 to SGM, R03AG073985 to KPL), the Conquer Cancer American Society of Clinical Oncology and Walther Cancer Foundation Career Development Award (to KPL), and the Wilmot Research Fellowship Award (to KPL). The information presented in this manuscript is solely the responsibility of the author(s) and does not necessarily represent the views of PCORI, its Board of Governors, or the Methodology Committee as well as the National Institutes of Health. We would like to thank Susan Rosenthal, MD, for her editorial assistance.

## CONFLICT OF INTEREST STATEMENT

KPL reported conflicts of interest outside of the submitted work: KPL has served as a consultant for Pfizer and Seagen and has received speaker fees from Pfizer. The remaining authors have no conflicts to report. RFD reported conflicts of interest outside of the submitted work: RFD has served on advisory boards for Exelixis Inc. and Merck & Co., and served as a consultant for Toray Industries, Inc.

## Data Availability

The data that support the findings of this study are available from the corresponding author upon reasonable request.
